# Automated Classification of Brain Tumors from Magnetic Resonance Imaging Using Deep Learning

**DOI:** 10.3390/brainsci13040602

**Published:** 2023-04-01

**Authors:** Zahid Rasheed, Yong-Kui Ma, Inam Ullah, Tamara Al Shloul, Ahsan Bin Tufail, Yazeed Yasin Ghadi, Muhammad Zubair Khan, Heba G. Mohamed

**Affiliations:** 1 School of Electronics and Information Engineering, Harbin Institute of Technology, Harbin 150001, China; 2 Department of Computer Engineering, Gachon University, Sujeong-gu, Seongnam 13120, Republic of Korea; 3 Department of General Education, Liwa College of Technology, Abu Dhabi P.O. Box 41009, United Arab Emirates; 4 Department of Computer Science, National University of Science and Technology, Balochistan Campus, Quetta 87300, Pakistan; 5 Department of Computer Science, Al Ain University, Abu Dhabi P.O. Box 112612, United Arab Emirates; 6 Faculty of Basic Sciences, BUITEMS, Quetta 87300, Pakistan; 7 Department of Electrical Engineering, College of Engineering, Princess Nourah bint Abdulrahman University, P.O. Box 84428, Riyadh 11671, Saudi Arabia

**Keywords:** brain tumors, magnetic resonance imaging, Deep Learning, neural network, tumor classification, healthcare, pre-trained models

## Abstract

Brain tumor classification is crucial for medical evaluation in computer-assisted diagnostics (CAD). However, manual diagnosis of brain tumors from magnetic resonance imaging (MRI) can be time-consuming and complex, leading to inaccurate detection and classification. This is mainly because brain tumor identification is a complex procedure that relies on different modules. The advancements in Deep Learning (DL) have assisted in the automated process of medical images and diagnostics for various medical conditions, which benefits the health sector. Convolutional Neural Network (CNN) is one of the most prominent DL methods for visual learning and image classification tasks. This study presents a novel CNN algorithm to classify the brain tumor types of glioma, meningioma, and pituitary. The algorithm was tested on benchmarked data and compared with the existing pre-trained VGG16, VGG19, ResNet50, MobileNetV2, and InceptionV3 algorithms reported in the literature. The experimental results have indicated a high classification accuracy of 98.04%, precision, recall, and f1-score success rate of 98%, respectively. The classification results proved that the most common kinds of brain tumors could be categorized with a high level of accuracy. The presented algorithm has good generalization capability and execution speed that can be helpful in the field of medicine to assist doctors in making prompt and accurate decisions associated with brain tumor diagnosis.

## 1. Introduction

A brain tumor is the growth of abnormal cells in the brain tissues. According to the World Health Organization (WHO), tumor is the second leading cause of mortality worldwide [1,2]. A brain tumor can be benign or malignant; unlike malignant tumors, benign tumors grow slowly, do not invade surrounding tissues or organs, and generally do not pose a serious threat to health. Benign tumors can be removed surgically and typically do not return after surgical removal [3]. Unlike benign tumors, malignant tumors invade surrounding tissues and organs and cause serious bodily harm if not treated promptly and effectively [4]. Therefore, early detection of brain tumors is very important to increase the survival of patients. The most common brain tumors are glioma, meningioma, and pituitary tumors. Glioma is a tumor that develops in the glial cells that surround and support neurons in the brain, including astrocytes, oligodendrocytes, and ependymal cells [5], pituitary tumor develops in the pituitary gland [6]; while meningioma forms in the meninges, which are the outer three layers of tissues between the skull [7]. The most distinguished contrast between these three tumors is that meningioma is commonly benign, whereas glioma is malignant, and the pituitary tumor is identified as benign [8].

Primary brain tumors can cause various symptoms depending on their size, location, and growth rate, regardless of whether the tumor is benign or malignant [9,10]. Furthermore, glioma may cause various symptoms, including aphasia, vision changes or loss, cognitive difficulties, and problems with walking or balance [11,12]. Meningioma typically has subtle symptoms that may gradually worsen, including changes in vision and morning headaches [13]. Pituitary tumors can result in headaches, vision problems, and double vision due to pressure on the optic nerve [6]. Therefore, distinguishing between these tumor types is crucial for clinical diagnosis and treatment evaluation. Early diagnosis of brain tumors largely depends on the expertise of radiologists. Magnetic Resonance Imaging (MRI) is commonly used to determine tumor types, but it relies on human interpretation and can be challenging to analyze large amounts of data [14]. The standard procedure for diagnosing and treating brain tumors, biopsies are seldom performed before conclusive brain surgery [15]. Developing a comprehensive diagnostics tool for tumor detection and classification from MR images is essential to acquire an exact diagnosis and prevent surgery and subjectivity [16]. Recent technological breakthroughs, particularly Artificial Intelligence (AI) [17] and Machine Learning (ML) [18,19,20,21,22], have had far-reaching implications on the healthcare sector, providing essential resources for several formerly ineffective healthcare sectors comprising imaging [23]. 

Various ML algorithms are determined for MR image detection and classification to give radiologists a new perspective. In addition to detecting tumors, medical imaging techniques are widely regarded as the most reliable and popular for diagnosing cancer in many forms. This method’s importance increases due to its lack of invasiveness [24,25,26,27,28,29]. Medical imaging techniques such as MRI are widely utilized because they provide clear pictures of brain tissue that can diagnose and classify different brain tumors. There is a vast variety of sizes, forms, and densities among brain tumors [30]. Moreover, tumors with distinct pathogenic features may appear identical. Many images inside the database created the most significant challenges when classifying the MR images using some neural networks. However, as MR images are obtained in different planes, using all of them might increase the database. Preprocessing is necessary before feeding the MR images into the various networks to achieve the classification result [24]. Convolutional Neural Networks (CNN) resolved this issue and has several benefits, including feature engineering, and preprocessing is not required. Utilizing a less complex network demands fewer resources for deployment and training. It is a major challenge due to the lack of resources to use the system for medical diagnostics or on mobile platforms. The method must be generally useful if it is required for daily routine clinical diagnostics. 

Our key contributions in this study are as follows:

- This study presents a novel CNN approach for classifying three types of brain tumors: glioma, meningioma, and pituitary tumors.
- The objective is to show that the presented approach can outperform more complex methods with limited resources for deployment and training. The study evaluates the network’s ability to generalize for clinical research and further deployment.
- The presented investigation suggests that the proposed methodology outperforms existing approaches, as evidenced by achieving the highest accuracy score on the Kaggle dataset. Furthermore, comparisons were made with pre-trained models and previous methods to reveal the prediction performance of the presented approach.

The following sections of this paper describe the literature in Section 2, the dataset, proposed architecture, pre-trained models, and optimization techniques in Section 3, the experimental results of the models in Section 4, and discussion in Section 5. The conclusion is presented in the last section.

## 2. Literature Review

Due to the above considerations, classifying brain tumors into discrete categories is arduous. MR image’s capacity to detect and classify brain tumors has been the subject of several studies that deployed various methodologies. Sasikala et al. [31] deployed wavelet-based feature extraction and a Genetic Algorithm (GA) to select features from brain tumors, and an artificial neural network was utilized for classification. EI-Dahshan et al. [32] classified the brain tumor using hybrid techniques; they extracted the features using Discrete Wavelet Transform (DWT), reduced the features using Principal Component Analysis (PCA), and then classified these features using Feedforward Backpropagation Artificial Neural Network (FP-ANN) and K-Nearest Neighbor (KNN) classifiers. 

Kaplan et al. [24] deployed distinct techniques, namely Local Binary Pattern (LBP), *n*LBP, and αLBP, for feature extraction, and the classification process was performed using K-Nearest Neighbor (KNN), ANN, Random Forest (RF), AIDE, and Linear Discriminant Analysis (LDA) methods; the highest success rate was achieved 95.56% with *n*LBP_d=1_ and KNN. Rathi and Palani primarily deployed the segmentation approach by applying several kernel-based probabilistic clustering algorithms on noise-free images filtered with a median filter [25]. The most significant features of the information acquired for each segment were evaluated using linear discriminant analysis, and Deep Learning (DL) based methods were utilized to categorize brain tumors. Mohsen et al. [33] investigated the application of Deep Neural Networks (DNNs) for classifying brain tumors. They tested the DNN-based classification system using 66 MR images of the brain and utilized extracted features using the discrete wavelet transformation and principal component analysis techniques. In 2015, Cheng developed a figshare dataset of brain tumors [34], and furtherutilized it [30] to attempt the three classes problem of detecting brain malignancies. Using image dilation, they magnified the tumor location and deployed Support Vector Machines (SVM) to classify the images after extracting the features using the intensity histogram, gray level co-occurrence matrix, and bag-of-words model; the highest classification results were achieved at 91.28%. Combining statistical features with the neural network method, Ismael and Abdel Qader [35] presented a framework for classification. Two-dimensional (2D), discrete wavelet transform, and 2D Gabor filter techniques were combined with supporting feature selection. Using a back propagation neural network as the classifier improved accuracy to 91.9% when testing the system on brain MRI data for cancer diagnosis. Abiwinanda et al. [36] utilized five diverse and straightforward CNN architectures and found that the two-layer convolution design achieved the best performance, with an accuracy rate of 84.19%. To classify brain tumors from MR images, Afshar et al. [14] utilized a modified CNN framework called Capsule network (CapsNet) and achieved a success rate of 90.89% for the classification. 

Pashaei et al. [37] extracted information from brain images using CNN, classified brain malignancies using Kernel Extreme Learning Machines (KELM), and achieved a 93.68% accuracy. According to Phaye et al. [38], multiple capsule networks were used to categorize brain cancers. This design improved the accuracy to 95.03% by replacing the standard convolution layer in the CapsNet with a densely connected convolution layer. Avşar and Salçin [39] applied DL to classify brain tumors and created a faster region-based CNN (Faster R-CNN) with a success rate of 91.66%. Zhou et al. [40] collected information from axial sections and obtained sequential information of many frames using dense CNN; for classification, they deployed a Recurrent Neural Network (RNN) and attained a 92.13% accuracy. Anaraki et al. [41] achieved an effective classification rate of 94.2% on brain tumor types, including glioma, meningioma, and pituitary, using a combination of CNN and GA as a classification technique. Gumaei et al. [42] deployed a hybrid feature extraction approach based on a Regularized Extreme Learning Machine (RELM) to enhance the accuracy of a classification method and achieved a success rate of 94.23%; RELM is used for classification after enhancing the contrast of brain edges and regions with the min–max normalization rule and extracted brain tumor features using the hybrid technique. Ghassemi et al. [43] deployed a DL classification system for brain tumors using a pre-trained Deep Neural Network (DNN) in a Generative Adversarial Network (GAN). The pre-training of the DNN was accomplished using multiple datasets to create features for the GAN. Following pre-training, the fully connected layers were swapped, and the resulting system achieved a success rate of 95.6% for brain tumor classification task. Swati et al. [44] implemented AlexNet, VGG16, and VGG19 with fine-tuning to classify brain tumors; the authors achieved 94.82% accuracy. Noreen et al. [45] used fined tuned models such as InceptionV3 and Xception to classify brain tumors, and the authors explored these models through ML algorithms such as softmax, random forest, SVM, K-nearest neighbors, and the ensemble techniques; they achieved the highest accuracy at 94.34% on ensemble InceptionV3. 

## 3. Material and Methods

**Figure 1:**
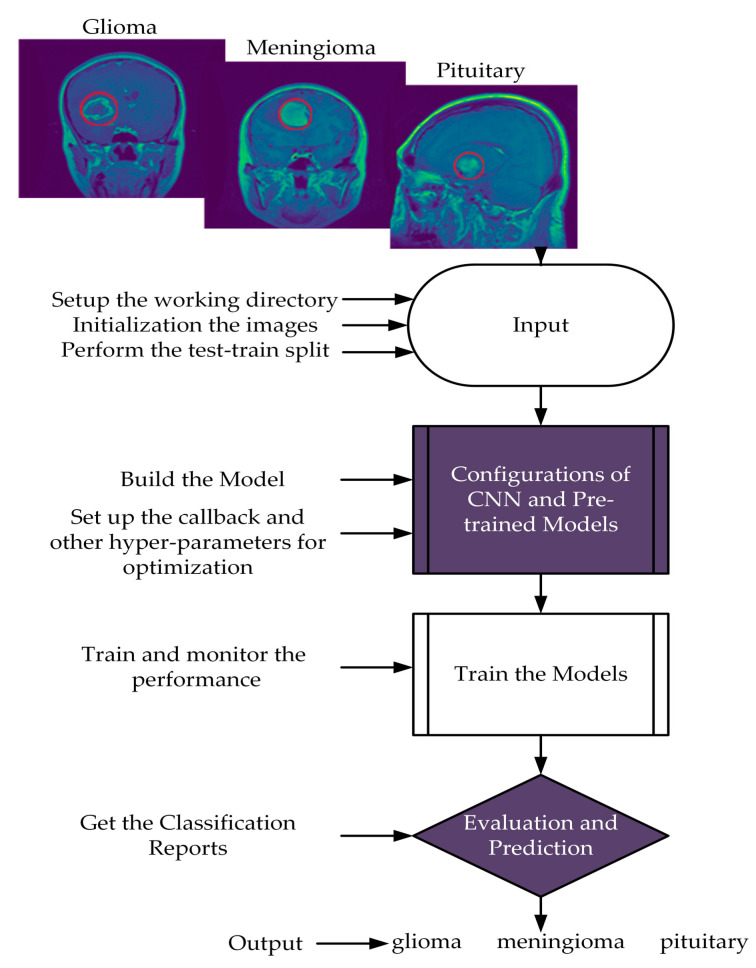
Flow chart of the proposed scheme.

This section presents the suggested scheme with the proposed CNN, which involves two major steps. Firstly, the input images were resized to maintain the same aspect ratio and normalized to preserve the uniform information distribution. The data were split into training 80% and testing 20% sets. Secondly, training approaches were performed on the training data to evaluate the presented model using Adam optimizer and ReduceLROnPlateau callbacks for learning rates. Furthermore, we evaluated the proposed model based on accuracy, precision, recall, and f1-score findings. The flow chart of the proposed scheme is illustrated in Figure 1. 

### 3.1. Dataset

This study utilized a dataset comprising 3064 T1 weighted contrast-enhanced MR images, which were acquired from two hospitals, namely Nangfang Hospital and General Hospital Tianjin Medical University, China. The images were collected between 2005 and 2010 and made available online in 2015. The most recent update to the dataset was performed in 2017 [34]. The dataset is also accessible on the Kaggle website in PNG format [46]. The collection consists of 233 patients, featuring three different tumor types: glioma (1426 images), meningioma (708 images), and pituitary (930 images). The images were captured in three different planes: sagittal (1025 images), axial (994 images), and coronal (1045 images), with original images of 512 × 512 dimensions. Figure 1 visually represents the various tumor forms in the dataset.

### 3.2. Network Architectures

#### 3.2.1. Proposed Model

**Figure 2:**
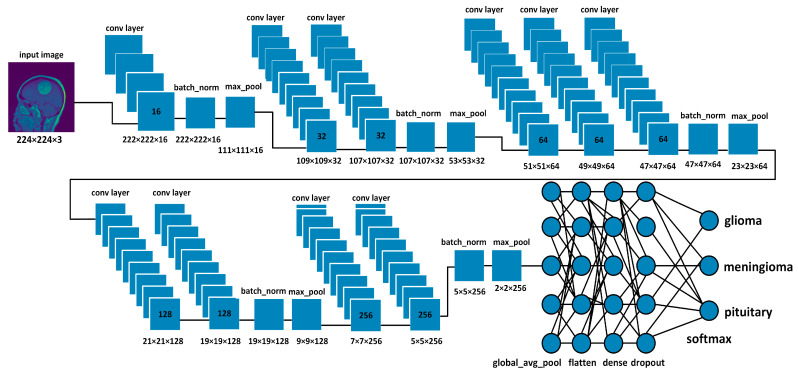
Proposed CNN architecture.

Figure 2 demonstrates the proposed CNN model; it extracted the MRI data with 224 × 224 input dimensions. We primarily used a single filter of 16 convolution layers [47] with a kernel size of 3 × 3, stride size of 1 × 1, and padding is valid. Subsequently, we used the batch normalization layer [48] and 2D max pooling layer of 2 × 2 to acquire maximum information on the images. In the same way, we added the number of convolution layers with the filter size of 32, 64, 128, and 256 having the same kernel size of 3 × 3, stride size of 1 × 1, and padding is valid. Subsequently, we applied the global average pooling [49], flattened, dense [50] (in the dense layer, we used 512 neurons and kernel regularizing techniques L1 (10^−5^) and L2 (10^−4^), and dropout [51] layers with 0.5%. In the end, the softmax function [47] was utilized with the output layer to determine the likelihood score for each class and classify the decision label as to whether the input image contained a glioma, meningioma, or pituitary tumor. 

Rectifier Linear Unit (ReLU) is the activation function employed for all convolutional layers; as demonstrated by Vinod and Hinton [52], it transforms the weighted input sum into the output of the nodes. The ReLU function can be mathematically represented as

(1) $$f\left(g\right)=max\;(0,g)$$

where g represents the input value when g is negative or equal to zero, the output is also zero. However, when g exceeds zero, the output is set to one. The ReLU function is frequently utilized in the hidden nodes of CNNs. The derivation of the function can be mathematically represented as

(2) $$f^{\prime }(g)=\left\{\begin{array}{c} 1,for\;g\geq 0\\ 0,for\;g<0 \end{array}\right.$$

In Equation (2), if the input value is zero, the corresponding neuron is considered “deceased” and will not be triggered. In addition, pooling layers are commonly used in CNNs to reduce feature maps’ spatial size (i.e., height and width) while retaining important information. It is important because as we move deeper into the neural network, the number of filters and feature maps increases, resulting in a high computational cost. Pooling layers help reduce the number of parameters in the model and prevent overfitting by reducing the spatial resolution. In max pooling, a fixed-size window slides over the input feature map and selects the maximum value within that window. The output of max pooling is a reduced-size feature map that highlights the most important features of the input. The max pooling operation can be defined as

(3) MaxPoolingxi,j=xi+m,j+nm,nmax

where x is the input feature map, i,j are the spatial coordinates of the output feature map, and m,n are the coordinates of the pooling window [47]. Global pooling is a type of pooling layer that takes the entire feature map as input and outputs a single value for each feature map. Global max pooling takes the maximum value of the feature map, while global average pooling takes the average value. Global pooling is useful when we want to reduce the dimensionality of the feature map and extract global information about the input. The global average pooling can be expressed as

(4) $$Gobalavgpooling\left(x\right)=\frac{1}{k\times 1}\sum_{i=1}^{k}\sum_{j=1}^{l}x_{i,j}$$

The equation for global average pooling operation on a feature map *x* with *k* channels and *l* spatial dimensions (height and width) the symbol ∑ represents the summation operation, *i* and *j* are the indices used to iterate over the spatial dimensions of the feature map, and *k* is the number of channels in the feature map. The result of the equation is a vector of *k* values, where each value represents the average activation for the corresponding channel across all spatial locations in the feature map. Furthermore, the loss function is used in DL to quantify the discrepancy between the algorithm’s predictions and the actual values. However, different optimization techniques can be employed to reduce the size of this error. This study used categorical cross-entropy for the loss function with softmax. In categorical cross-entropy, the error rate is calculated by using Equation (5); mathematically expression follows as

(5) $$L_{CE}=-\sum_{j}y_{i,j}log({\hat{y}}_{i,j})$$

**Figure 3:**
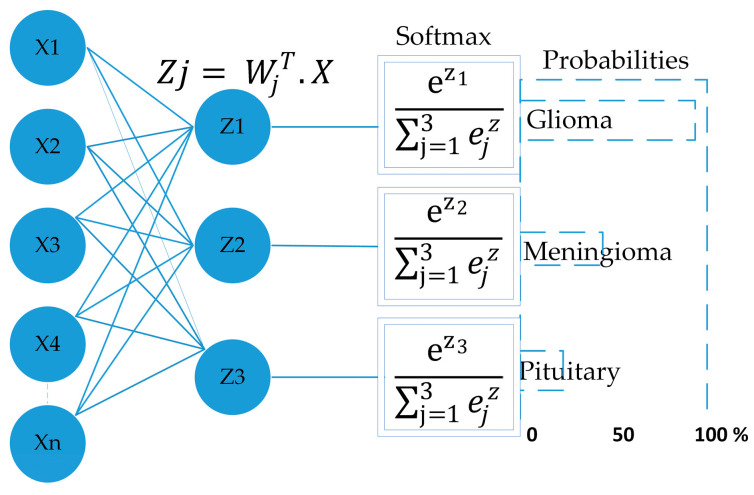
Shows the softmax function as the output layer in a neural network, where the input vector x is transformed through hidden layers to produce an output vector z, representing the scores for each class. The softmax function is then applied to z to obtain a probability distribution over the classes.

In Equation (5) where $L_{CE}$ are samples of loss value, i is the $i^{th}$ sample in the set j is the label/output index, y is the actual value, and $\hat{y}$ is predicted value. Furthermore, the softmax function is used as an output layer to normalize the model output into a probability distribution over predicted output classes. The following equation shows the function of softmax.

(6) $$\sigma {\left(\vec{Z}\right)}_{i}=\frac{e^{Z_{i}}}{\sum_{j=1}^{K}e^{Z_{j}}}$$

where σ represents softmax, $\vec{Z}$ denotes the input vector, $e^{Z_{i}}$ represents the standard exponential function of the input vector, *K* represents the number of classes, $e^{Z_{j}}$ represents the standard exponential function of the output. Figure 3 depicts the function of softmax as the output layer [47].

#### 3.2.2. Optimization Approaches

Several optimization strategies are used to decrease the loss in deep neural networks by adjusting parameters such as weights and learning rates. This study used Adam optimizer regularization methods, dropout, and ReduceLROnPlateau callbacks. The adaptive moment estimation (Adam) optimizer was developed by Diederik Kingma [53]. Adam is an optimizer that uses RMSprop in conjunction with a stochastic gradient descent algorithm based on momentum. Herbert and Sutton [54] suggested the stochastic gradient descent method. The pseudocode of the Adam algorithm (Algorithms 1) is given as below.
**Algorithm 1:** Pseudocode: For the Adam algorithm.
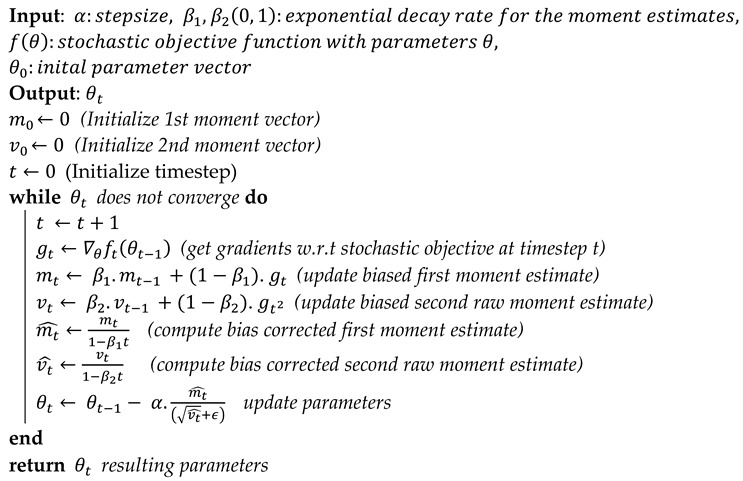


Regularization is a group of methods that can avoid overfitting in neural networks and, as a result, improve the accuracy of a DL model when presented with new data from the problem domain [54,55]. L2 and L1 are standard and effective regularization techniques used in our model. L2 regularization is known as weight decay or ridge regression, and L1 is known as Lasso regression. The cost function can be calculated by using the following equations.

(7) $$L1\;Cost\;fuction=cost\;funtion\left(Loss\right)+\lambda \sum_{i=1}^{N}\left|w_{i}\right|$$

(8) $$L2\;Cost\;fuction=cost\;funtion\left(Loss\right)+\lambda \sum_{i=1}^{N}\left|w_{i}^{2}\right|$$

**Figure 4:**
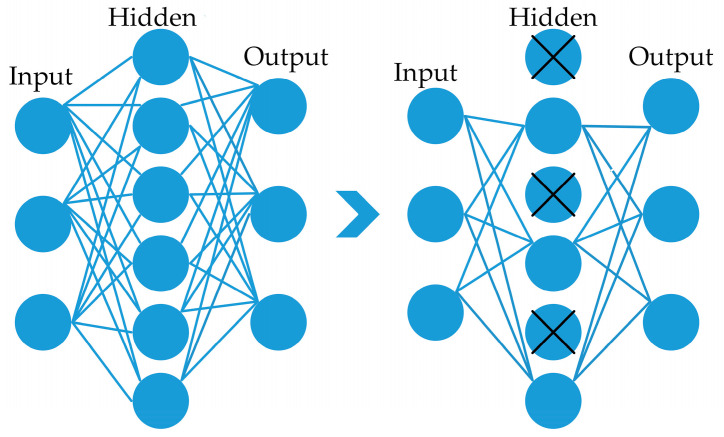
Depiction of a dropout layer with a rate of 0.5% on the right side.

The equations involve a hyperparameter denoted by λ, which controls the strength of regularization, *N* is the number of model parameters, and $w_{i}$ represents the *i^th^* parameter, ∑ the sum of all parameters. Dropout is also a regularization technique intended to enhance the ability of a network to generalize and prevent it from becoming very proficient at its task. Typically, a dropout [56] value between 0.2 and 0.5 is employed; if the dropout probability value is too low, it is of little consequence. However, if the value is too high, the network might not learn enough about the features during model training. This layer eliminates the random activation nodes, significantly boosting the training phase. In the proposed framework, 0.5% of dropouts found a suitable dropout value, as an example is shown in Figure 4.

Callbacks: In model training, we used ReduceLROnPlateau [57] callbacks. ReduceLROnPlateau callback in Keras can be used to reduce the learning rate of the model during training if the validation loss has stopped improving. By reducing the learning rate, the optimization process can make smaller steps toward the minimum of the loss function, which can help the model become more efficient. It is also worth mentioning that the ReduceLROnPlateau callback works by keeping track of the best-observed value of the monitored quantity and reducing the learning rate when the current value has not improved for a certain number of epochs. A factor is used to reduce the learning rate; the following equation represents the new learning rate using a factor.

(9) new_learning_rate=learning_rate(0.001)∗factor(0.4)

The factor value should be between 0 and 1; if the value exceeds 1, the learning rate will explode. If the factor is 1, the learning rate would never decay. 

### 3.3. Pre-Trained Models

Pre-trained models are ML models trained on large-scale datasets such as ImageNet, which contains a million images from different classes and can be used for image classification, object detection, and other tasks. The idea behind pre-trained models is that they have already learned to recognize patterns in the data to be used as a starting point for a new task rather than training a model from scratch. Five pre-trained models, including VGG16, VGG19, InceptionV3, ResNet50, and MobileNetV2, were utilized in this study.

#### 3.3.1. VGG16

VGG16 was proposed in 2014 by Karen Simonyan and Zisserman [58] of Oxford University’s Visual Geometry Group. The architecture consists of 16 layers, including 13 convolutional layers, 3 fully connected layers, and small filters of size 3 × 3 with a stride size of 1. The max pooling layers use a 2 × 2 pooling window with a stride size of 2. It has 138 million parameters and is widely used for feature extraction in transfer learning.

#### 3.3.2. VGG19

VGG19 [58] is an extension of the VGG16 architecture; it has 19 layers, including 16 convolutional layers, 3 fully connected layers, and a small filter of size 3 × 3 with a stride size of 1. It also uses max-pooling layers with a 2 × 2 pooling window and a stride size of 2. It has 144 million parameters, more than VGG16, making it more powerful but computationally expensive. 

#### 3.3.3. ResNet50

Deep neural networks perform better as the model’s depth increases, which has been proven in the literature [59,60]. As the network size increases, vanishing/exploding gradients become problematic. To address this issue, the authors of ResNet50 [61] deployed a residual module, allowing the network to learn the residual mapping between the inputs and outputs rather than the original mapping. It is achieved by adding shortcut connections that bypass certain layers and adds the input to the output of the modules. The residual blocks help to alleviate the vanishing gradient problem and avoid degradation as the network depth increases. The ResNet50 is an architecture that uses a combination of convolutional layers with varying filter sizes (1 × 1, 3 × 3, 1 × 1) within bottleneck blocks, along with max pooling and average pooling layers to extract the features from the input images. 

#### 3.3.4. InceptionV3

The inception model [62] is offered in three different versions, each of which improves upon its successors in one or more ways. This one is quite complex compared to the previous version, which consisted of stacked layers. The engineering behind it allows it to operate more quickly and accurately. This strategy provides an advantage by deploying several kernel sizes at the same level, making the network wider rather than deeper. The authors created a single module by merging a max pooling layer at the same level with kernel sizes of 1 × 1, 3 × 3, and 5 × 5. The output results would be concatenated before forwarding since adding all of these layers at once would increase the computational demands of this model. To alleviate this, the authors included a 1 × 1 convolution layer before the 3 × 3 and 5 × 5 layers and after the max pooling layer. This layer uses 1 × 1 layers instead of 5 × 5 layers to save computing by reducing the number of input channels [62]. 

#### 3.3.5. MobileNetV2

The architecture was designed for mobile and embedded applications to achieve high accuracy while being lightweight and efficient in computation and memory usage. The model uses inverted residual, linear bottlenecks, and width multiplier parameters. The inverted residual is a series of convolutional layers that increase network capacity while minimizing computation and memory usage by expanding the input to a large number of channels, then convolving with a small kernel, and finally projecting back to a smaller number of channels. Linear bottlenecks reduce the number of parameters required by using a linear activation function instead of a nonlinear one. The width multiplier parameter scales the number of channels in the network [63].

## 4. Experimental Results

**Figure 5:**
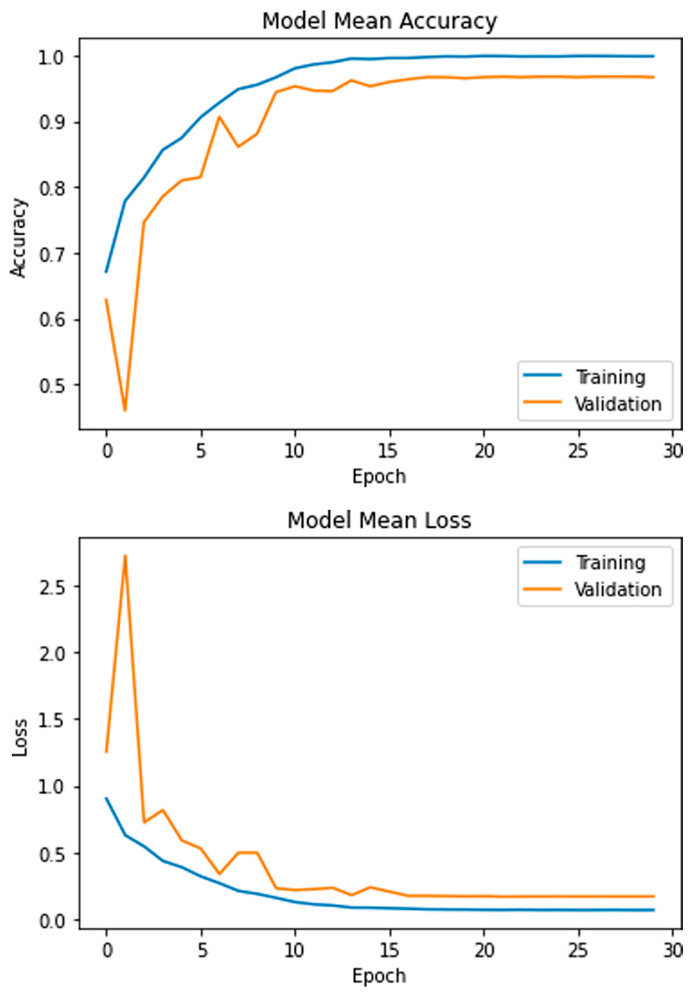
Mean accuracy and losses of the presented model.

The aim of this study is to classify the MRI dataset containing 3064 images of the glioma, meningioma, and pituitary tumors using the proposed model. Initially, the dataset was resized and separated into training and testing sets. In all experiments, the data were shuffled using a random state value of 101. The model was trained for 30 epochs using five-fold cross-validation and a batch size of 8 on the Adam optimizer. The learning rates were optimized with the ReduceLROnPlateau callbacks. The mean accuracy and losses of the presented model are presented in Figure 5. During the initial training phase, the graphs exhibit fluctuations, which can be attributed to the utilization of the ReduceLROnPlateau callback. This callback dynamically adjusts the optimizer’s learning rate during training based on the plateauing of the loss function. Following the 15th epoch of training, the optimizer is observed to converge more smoothly to an optimal set of weights, reducing the fluctuations of the accuracy and loss curves.

The platform utilized several libraries, including TensorFlow, Keras, Pandas, Numpy, Matplotlib, and Sklearn, to facilitate the data and model-building processes. The Central Processing Unit (CPU) used was an Intel(R) Core(TM) i7-7800 with a processing speed of 3.5 GHz. The Graphical Processing Unit (GPU) used was an NVIDIA GeForce GTX 1080 Ti, which enabled efficient model training and optimization. The software employed for the study was Python 3.7, which provided a comprehensive set of tools for data manipulation, analysis, and visualization. The platform had a total RAM capacity of 16 GB, sufficient for handling the data used in the study.

### 4.1. Evaluation Matrix

The proposed framework’s accuracy, precision, recall, and f1-score were evaluated. Recall measures the model’s ability to accurately identify the correct type of tumor, calculated as the ratio of true positives to the sum of true positives and false negatives. Precision measures the model’s ability to avoid misclassifying negative examples as positive and is calculated as the ratio of true positives to the sum of true and false positives. The f1-score is the harmonic mean of precision and recall and is calculated as two times the product of precision and recall divided by their sum. Accuracy measures the model’s overall performance in correctly classifying and is calculated as the ratio of correct predictions to the total number of predictions. The mathematical expressions for recall, precision, f1-score, and accuracy are represented by Equations (10)–(13) [64].

(10) $$Recall=\frac{TP}{TP+FN}$$

(11) $$Precision=\frac{TP}{TP+FP}$$

(12) $$f1\text{-}Score=2\times \frac{Recall\times Precision}{Recall+Precision}$$

(13) $$Accuracy=\frac{TP+TN}{TP+TN+FP+FN}$$

**Figure 6:**
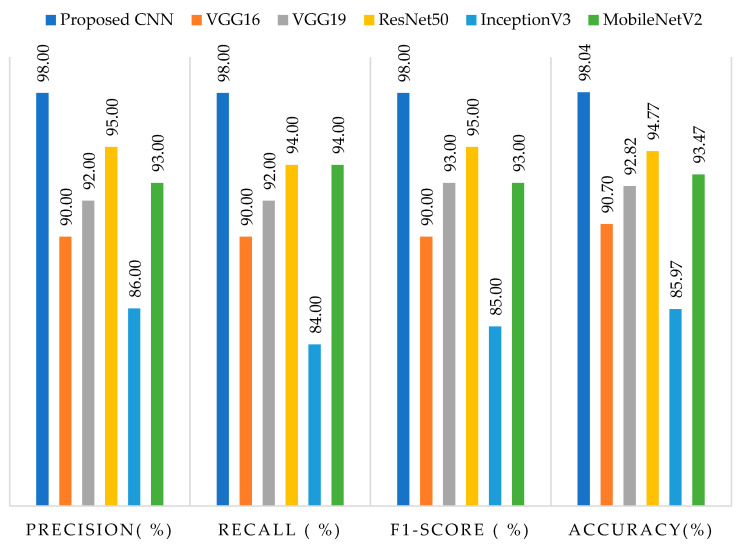
Represents the precision, recall, f1-score, and accuracy results on testing data with the proposed framework and pre-trained models.

The results of average precision, recall, f1-score, and accuracy on testing data for both the suggested framework and pre-trained models are presented in Figure 6. The proposed model achieved the highest accuracy rate of 98.04%, as well as precision, recall, and f1-score rates of 98%, while InceptionV3 exhibited the lowest performance, with an accuracy rate of 85.97%, precision rate of 86%, recall rate of 84%, and f1-score rate of 85%. It is worth noting that the inferior performance of InceptionV3 could be attributed to the utilization of multiple parallel convolutional and pooling layers, which are not well-suited for small datasets, as corroborated by our findings. Among the pre-trained models, ResNet50 demonstrated superior accuracy, precision, recall, and f1-score rates compared to VGG16, VGG19, and MobileNetV2. Moreover, default input sizes of 224 × 224 were employed for VGG16, VGG19, ResNet50, and MobileNetV2, whereas InceptionV3 employed 299 × 299 as its input size.

### 4.2. Confusion Matrix

**Figure 7:**
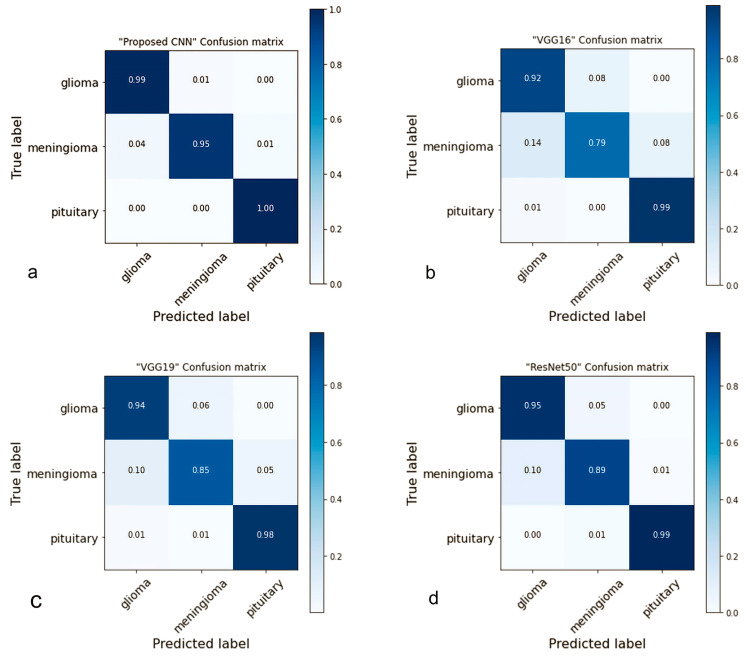

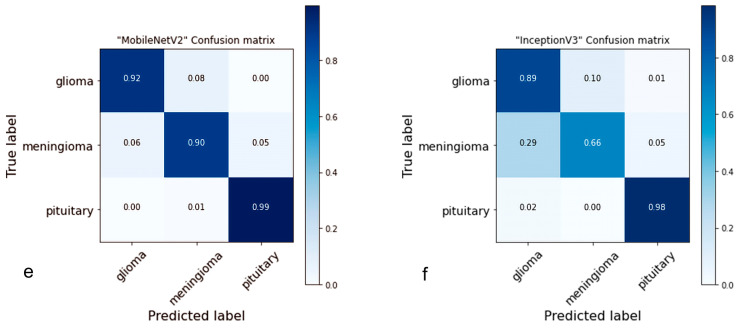
The confusion matrices of the proposed and pre-trained models on the testing data are presented in Figure 7. The figure displays the prediction rate of each model. Specifically, (**a**) illustrates that the proposed model achieved a high accuracy rate of 98.04%. Comparatively, (**b**) shows that VGG16 obtained an accuracy rate of 90.70%, (**c**) reveals that VGG19 achieved 92.82%, (**d**) demonstrates that the accuracy rate of ResNet50 was 94.77%, (**e**) indicates that MobileNetV2 achieved 93.47%, and lastly, (**f**) depicts that InceptionV3 achieved an accuracy rate of 85.97%.

A confusion matrix is a table used to evaluate the performance of classification models [65]. The proposed network performed well in multi-tumor classification and properly detected each type of brain tumor in this investigation. Figure 7 illustrates the results obtained from the testing data, which can be compared with pre-trained models and had low performance compared to the suggested models. In comparison, the proposed model accurately predicted glioma 99%, meningioma 95%, and 100% pituitary; the predicted ratio was greater than the pre-trained models. In addition, the meningioma success rate was not very high in this study; we consider it for further studies.

In addition, ResNet50 predicted (glioma 95%, meningioma 89%, and 99% pituitary) was a better success rate compared to VGG19 predicted (glioma 94%, meningioma 85%, and 98% pituitary), VGG16 predicted (glioma 92%, meningioma 79%, and 99% pituitary), InceptionV3 predicted (glioma 89%, meningioma 66%, and 98% pituitary) and MobileNetV2 predicted (glioma 92%, meningioma 90%, and 99% pituitary). 

### 4.3. ROC Curve Analysis

**Figure 8:**
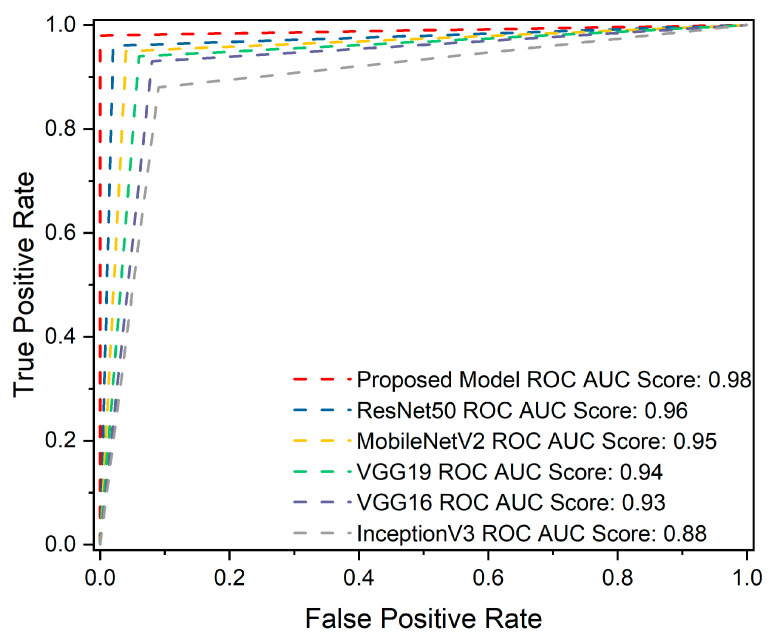
The overall receiver operating characteristic (ROC) area under the curve (AUC) score for the proposed and pre-trained models. The proposed model obtained the highest ROC AUC score of 98%, indicating its superior ability to discriminate between positive and negative classes. In contrast, ResNet50 scored 96% in ROC AUC, MobileNetV2 achieved a score of 95%, VGG19 scored 94%, VGG16 scored 93%, and InceptionV3 achieved 88%. These results demonstrate the superiority of the proposed model over the pre-trained models in terms of ROC AUC scores, underscoring its robustness in differentiating among the brain tumor classes.

The Receiver Operation Characteristics (ROC) curve is crucial for identifying brain tumors. True Positive Rate (TPR) and False Positive Rate (FPR) [66] are two metrics through which the prediction performance can be calculated at all classification thresholds on testing data. In comparison, the proposed model predicted (glioma 0.98%, meningioma 0.97%, pituitary 1.00%), ResNet50 predicted (glioma 0.95%, meningioma 0.93%, pituitary 0.99%), MobileNetV2 predicted(glioma 0.95%, meningioma 0.92%, pituitary 0.99%), VGG19 predicted (glioma 0.94%, meningioma 0.91%, pituitary 0.98%), VGG16 predicted (glioma 0.93%, meningioma 0.87%, pituitary 0.98%), and InceptionV3 (glioma 0.87%, meningioma 0.80%, pituitary 0.98%). Figure 8 demonstrates the overall ROC AUC scores.

It is common practice in the literature to use hyperparameters to optimize the learning process during training. This work employed ReduceLROnPlateau callbacks with the Adam optimizer because the Adam algorithm uses the stochastic gradient method to update the weights of a neural network during training. It adapts the learning rate for each parameter based on the estimates of the gradient’s first and second moments, which can lead to faster convergence and better performance. ReduceLROnPlateau callback reduces the learning rate when a matric (e.g., validation loss) has stopped improving. It helps the model avoid getting stuck in a suboptimal local minimum [67] and can result in better generalization and lower test error. The five-fold cross-validation method [68] randomly divides the data into five subsets and trains and assesses the model five times; these five runs provide a more accurate assessment of the model’s performance on test data than a single split. Using these techniques together was helpful in model training with the best results. In comparing training and testing time for each epoch using 8 batch size, the proposed model grabbed less time, specifically 12 ms/step. In contrast, VGG16 grabbed 24 ms/step, VGG19 30 ms/step, ResNet50 28 ms/step, MobileNetV2 16 ms/step, and InceptionV3 grabbed 34 ms/step.

## 5. Discussion

This study presented a classification approach for primary brain tumor types such as glioma, meningioma, and pituitary by applying a CNN model to MR images. Table 1 summarizes the findings from previous research involving the same types of brain tumors but with different methods. The reliability of the proposed system is demonstrated by the fact that the proposed structure provides the most precise prediction results compared to previous studies of a similar nature. The suggested CNN method is a segment-free approach, as the brain tumor images are loaded to obtain classes of tumors, unlike the other methods that require additional manual processes such as feature extractions or localization of tumors. For example, ref. [35] extracted the features with DWT and the Gabor filter and then deployed them in another stage for classification [14]. The authors utilized coarse boundaries as an additional input to aid the network in producing better classification outcomes. Ref. [37] deployed CNN for feature extraction and then classified by KELM, while ref. [39] used R CNN to extract features from MR images and then used these features to classify the tumors [40]. They collected characteristics from axial slices using DesnesNet and used these features for classification [41]. The authors used GA to optimize the CNN structure for the best classification accuracy [42]. They retrieved the feature using PCA-NGIST methods and then used these features for classification. However, the proposed model achieved a favorable classification rate without the preceding stage, demanding another manual process to localize the tumors before training. Furthermore, the proposed model employed the ReduceLROnPlateau callbacks that automatically adjust the learning rate without manually tuning the learning rate schedule; finding the optimal learning rate can be challenging and time-consuming [43,44,45]. The authors used pre-train approaches to solve the problem, which were not precise predictions compared to our proposed framework.

## 6. Conclusions

This study presented a convolutional neural network (CNN) that can accurately classify various types of brain tumors, such as glioma, meningioma, and pituitary tumors. We compared the performance of our proposed model with previous and several pre-trained models, namely VGG16, VGG19, ResNet50, MobileNetV2, and InceptionV3. Our findings suggest that the presented model exhibits superior accuracy of 98.04%, generalization capability, and execution speed, which makes it a valuable decision-support tool for routine clinical diagnostics. Moreover, the proposed method can contribute significantly to the early identification of life-threatening illnesses in various clinical domains, including medical imaging, where lung and breast cancer is associated with high mortality rates worldwide. For future work, we intend to explore data augmentation techniques to increase the number of images, thereby improving the generalization capability of the networks. Additionally, we plan to develop real-time detection systems for brain tumors in the operation room and 3D networks for other medical images.

## Figures and Tables

**Table 1:** Comparison between the proposed model and previous related work.

Authors	Methods	Average Precision	Average Recall	Average F1-Score	Accuracy
Ismael and Abdel-Qader [35]	DWT-Gabor-NN	X	X	X	91.9
Afshar [14]	CapsNet	X	X	X	90.89
Pashaei [37]	CNN + KELM	94.6	58.43	93	93.68
Avşar and Salçin [39]	R-CNN	97	X	95	91.66
Zhou [40]	LSTM + DenseNet	X	X	X	92.13
Anaraki [41]	CCN + GA	X	X	X	94.20
Gumaei [42]	Hybrid PCA-NGIST + RELM	X	X	X	94.23
Ghassemi [43]	CNN based GAN	95.29	X	95.10	95.60
Swati [44]	VGG16 Finetune	89.17	X	91.50	94.65
Swati [44]	VGG19 Finetune	89.52	X	91.73	94.82
Swati [44]	AlexNet	84.56	X	86.83	89.95
Noreen [45]	InceptionV3 Ensemble	93	92	92	94.34
Our studies	Proposed CNN	98	98	98	98.04

## Data Availability

No new data were created or analyzed in this study. Data sharing is not applicable to this article.
